# Mastication-Enhanced Taste-Based Classification of Multi-Ingredient Dishes for Robotic Cooking

**DOI:** 10.3389/frobt.2022.886074

**Published:** 2022-05-04

**Authors:** Grzegorz Sochacki, Arsen Abdulali, Fumiya Iida

**Affiliations:** Bio-Inspired Robotics Laboratory, Department of Engineering, University of Cambridge, Cambridge, United Kingdom

**Keywords:** electronic tongues, mastication, robotic chef, robotic cooking, taste feedback, salinity sensing, conductance sensing

## Abstract

Chefs frequently rely on their taste to assess the content and flavor of dishes during cooking. While tasting the food, the mastication process also provides continuous feedback by exposing the taste receptors to food at various stages of chewing. Since different ingredients of the dish undergo specific changes during chewing, the mastication helps to understand the food content. The current methods of electronic tasting, on the contrary, always use a single taste snapshot of a homogenized sample. We propose a robotic setup that uses the mixing to imitate mastication and tastes the dish at two different mastication phases. Each tasting is done using a conductance probe measuring conductance at multiple, spatially distributed points. This data is used to classify 9 varieties of scrambled eggs with tomatoes. We test four different tasting methods and analyze the resulting classification performance, showing a significant improvement over tasting homogenized samples. The experimental results show that tasting at two states of mechanical processing of the food increased classification F1 score to 0.93 in comparison to the traditional tasting of a homogenized sample resulting in F1 score of 0.55. We attribute this performance increase to the fact that different dishes are affected differently by the mixing process, and have different spatial distributions of the salinity. It helps the robot to distinguish between dishes of the same average salinity, but different content of ingredients. This work demonstrates that mastication plays an important role in robotic tasting and implementing it can improve the tasting ability of robotic chefs.

## 1 Introduction

Culinary arts is one of the activities where human dominates over automated robotic systems with a great advantage. Automation of culinary tasks necessitates solving challenges in many fields. Some of these challenges were tackled, including translation of recipe Beetz et al. (2011) and human chef body pose Danno et al. (2021) into robotic action. Few attempts to build commercial robotic kitchens were made, including integrating robots into kitchens (Moley Robotics, 2022) and launching a robotic restaurant (Spyce Ltd, 2021). Visual feedback was used to adjust frying time of a sausage, but only simple and not robust approach of background masking and averaging the hue was used Mauch et al. (2017). Teleoperation was also used to help the robotic chef at cake decoration Bolano et al. (2019). Loading dishwashers with robotic arms was also investigated Voysey et al. (2021). Some robotic chefs can improve their cooking based on a feedback from diners Junge et al. (2020) or replicate a human cooked dish using its own taste Sochacki et al. (2021).

However, one of the most influential differences between the cooking procedure of robotic and human chefs is that the latter continuously taste the food during the cooking. Moreover, the chewing process enhances tasting, as in addition to the flavour of a bite, the mastication enables tasting flavour changes during the mechanical processing of the food. An example of such flavour changes is chewing a tomato, causing it to release the juice, and changing the perceived taste. Saliva release is also a part of mastication. It is causing at least two types of effects–wetting the food and introducing digestive enzymes. Examples of these effects are melting of a sugar cube inside a mouth, which releases a strong sweet taste, as well as sweet taste arising after keeping bread in the mouth for a moderately long time–effect of exposure to enzymes from saliva. All these effects take a part in the tasting process and need to be understood to match the human ability to taste with a robotic setup.

While few electronic tongue implementations are proven effective Di Rosa et al. (2020), and some are even commercially available ASTREE (2022), Insent (2022), they all require precise and elaborate preprocessing for any non-liquid samples. For example, e-tongue was used for detection of meat adulteration, but the samples required 3 min of mincing Tian et al. (2019). Similarly, tracking the taste of Dezhou-Braised Chicken required homogenization with distilled water and centrifugation Liu et al. (2017). Other examples include mixing cheese samples with distilled water for 10 min Valente et al. (2018), while in the case of cheddar analysis the sample was homogenized with chloroform, before the addition of methanol and water and waiting for 20 min Lipkowitz et al. (2018). Some liquids like honey and sugary syrups also required dissolving in water and 10 min wait before tasting Oroian et al. (2018). Classification of oils was also done, but the samples needed to be mixed with distilled water, alcohol and mixed for 10 min Dias et al. (2014). Voltammetric sensors were used for classification of grapes, but required freshly squashed must to be prepared Rodriguez-Méndez et al. (2014). Similarly, mandarin quality was assessed with e-tongue based on its juice Qiu et al. (2015). Other studies limit themselves to liquid samples Zhang et al. (2019).

Equipment necessary for discussed preprocessing prohibits its application in kitchens. Furthermore, the required time delays the feedback, effectively making it impossible to react in time when cooking. Therefore, existing solutions are not compact and fast enough for robotic chef applications. Furthermore, the tendency to produce a wet homogeneous pulp for the sensor trivializes a large part of a human experience of tasting, and human’s agency over the measurement result–mastication, tongue movement, saliva production and more. Noticeably, all the spatial structure of the dish is completely lost. Additionally, the whole time sequence of measurements usually available for a human taster across stages of mastication is reduced to a single measurement, effectively removing the time dimension of the tasting experience.

In this paper, we developed a robotic setup reproducing the human mastication process to extract additional time-variant information. We show that tasting at several states of mechanical processing of the food can significantly increase the classification performance of the food with different amounts of the same ingredients. To prove the concept, we built a robotic setup equipped with a salinity sensor for taste measurements. We prepared the sample set of nine dishes of scrambled eggs with tomato, containing different quantities of tomato and salt in each dish. The experimental results show that tasting at even two states of mechanical processing of the food increased classification F1 score to 0.93 in comparison to the traditional tasting of a homogenized sample resulting in F1 score of 0.55. We also highlight how different compositions of a dish can result in the same measurement outcomes after homogenization, therefore making the classification task impossible.

## 2 Model of Tasting

**FIGURE 1:**
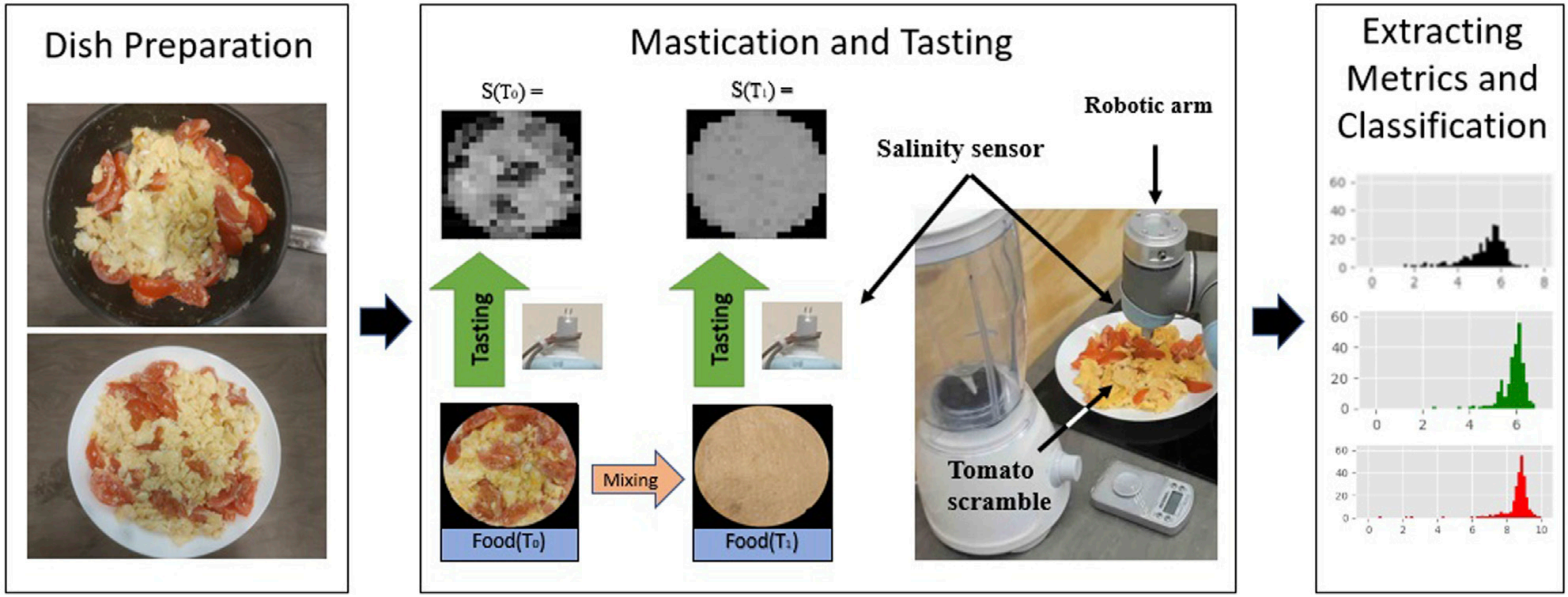
Experiment overview. Nine dishes is prepared for robotic tasting. Each of the dishes is tasted by the robot before and after mixing. A set of taste metrics is then extracted from each tasting and used to train a test SVM classifier.

We model the tasting process as a series of measurements at different moments in time and stages of chewing. Moreover, we take into account the fact that the human tongue has multiple receptors distributed across its surface. This fact is represented in the experiment by tasting in multiple spots and representing flavour as an array of measurements. The robot used is fitted with a single salinity sensor. To mitigate this limitation we fit the sensor on a robotic arm and physically move the sensor to multiple spots. Furthermore, the known location of samples enables showing the data as an image (further referred to as a taste map). This approach is shown in the middle of Figure 1. The taste map is further shown in Figure 3.

Mastication is the process of crushing and grinding food and its main purpose is to reduce the average size of the food particle. Smaller particles present a larger surface area for digestive enzymes to act. However, chewing also plays an important part in the tasting process, as the flavour changes while chewing. Our setup simulates it with a mixer shown in Figure 1.

Every tasting model needs a computation component to produce a meaningful signal Di Rosa et al. (2020), otherwise, it remains a collection of measurements. In our application, we use previously established taste metrics Sochacki et al. (2021) to reduce the dimensionality of data. These taste metrics relate to the average salinity and “mixed-ness” of a dish. Various subsets of these samples are then used to train and validate the support vector classifier. Support vector machines were previously used for taste-based classification with success Ouyang et al. (2013), Zhang et al. (2019). This computation part of sensing is represented by the right part of Figure 1.

### 2.1 Salinity Sensing

The robot recreates salinity taste with a conductance sensor. A conductance sensor determines the degree of ease for current to flow through a sample. This is achieved by measuring the current flowing through the sample between two electrodes, under pre-determined voltage. The dominant mechanism behind the conductivity is the movement of ions, therefore the salinity increases with ion concentration, ion mobility and the ionic charge. Due to low cost, robustness and ease of use, salinity sensors are a prime candidate for robotic applications. Conductance sensing is also used in the food industry, for example, to detect milk adulteration Sadat et al. (2006) and determination of salt content in different foods Benjankar and Kafle (2021).

For the need of this experiment, we created a salinity sensor probe made out of a testing tip of a standard salinity sensor testing device (ExTech salinity probe) same as in our previous work Sochacki et al. (2021). The same calibration procedure was followed, as well as placing the sample on a non-conductive (ceramic) plate. The main differences between this implementation and the human taste of saltiness are the lack of ion specificity (saltiness is selective for Na+ and K+ ions) and the ability to pierce the sample (tongue is limited to tasting the surface).

Moreover, the contact between electrodes and the dish can influence the reading. For example, piercing the dish at different depths will result in conduction paths of different depths, therefore different conductances. This effect was mitigated by always piercing at the full length of the electrode, resulting in conduction paths of the same depth for all the samples. Temperature can also affect the conductance, therefore the dish was always chilled down to room temperature before tasting.

## 3 Experimental Setup and Procedures

### 3.1 Robotic Setup

**FIGURE 2:**
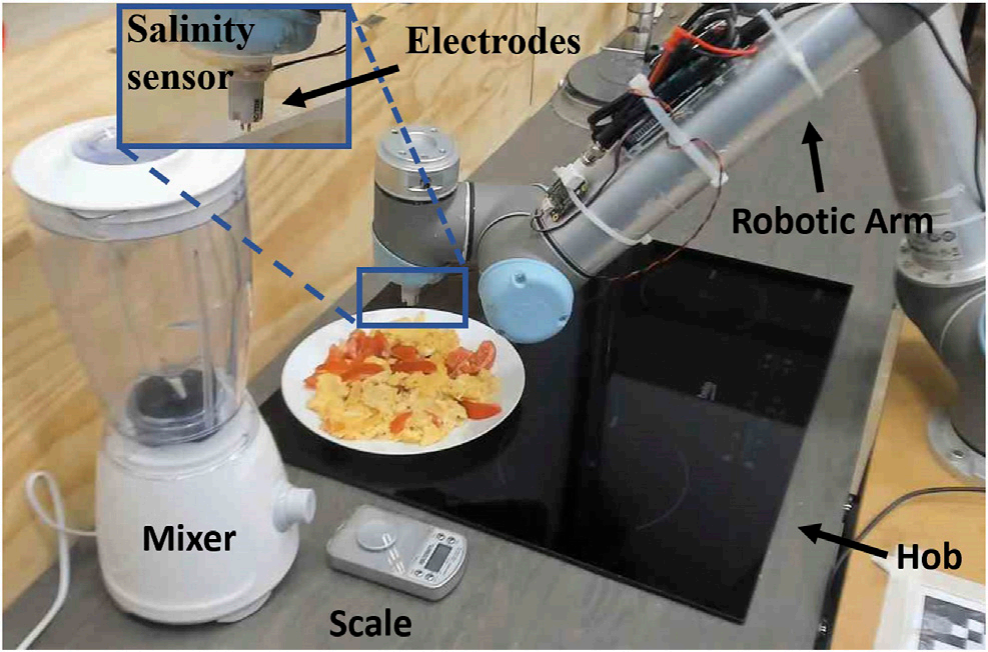
Experimental setup. UR5 robot is fitted with conductance sensor for saltiness tasting. Induction hob is used for cooking. Food is presented for tasting on a ceramic plate. The whole setup is controlled by a program run on a laptop.

The setup for the experiment, shown in Figure 2, consists of a UR5 robotic arm fitted with a conductance-based taste sensor. The sensor is placed in a place of an effector and is controlled by Arduino UNO, which provides an interface to a laptop *via* USB. The sensor is able to achieve 2 Hz sampling rate, including all interfacing to the laptop and saving the data. UR5 arm is also controlled by the laptop, effectively making the laptop a centre of the whole system. The robotic arm is placed on a trolley, allowing its convenient placement in the kitchen. Python program is used to process information from the sensor, store it, and analyze it. Dishes are prepared using a pan on an induction hob. A prescribed amount of salt is measured using a scale with an accuracy of 0.05 g. A porcelain plate is used as a waterproof and non-conductive platform for the tasted dish. Mastication is recreated using a mixer.

### 3.2 Ingredients and Produce

The cooked dishes were made of three products only, leaving out ingredients like butter to make the dishes more consistent. We use large free-range eggs (The Co-operative Group; United Kingdom) for the experiment. We weight and measured with a calliper 12 of them, obtaining an average weight of 68.1 g with a standard deviation of 2.37 g, an average diameter of 45.6 mm with a standard deviation of 0.72 mm, as well as an average height of 58.3 mm with a standard deviation of 1.03 mm. All tomatoes come from a local store (The Co-operative Group; United Kingdom). These are vine tomatoes rated as class 1, grown in Italy, and are of a standardized size (radius between 47 and 67 mm). We weight and measured with a digital calliper 12 of them, obtaining an average weight of 82.1 g with a standard deviation of 19.57 g, and an average diameter of 53.4 mm with a standard deviation of 3.46 mm, as well as an average height of 47.8 mm with a standard deviation of 4.05 mm. All of the measurements are presented in concise form in Table 1. We use standard table salt (The Co-operative Group; United Kingdom), purchased in a large bottle.

**TABLE 1:** Table showing the distribution of size and weight of the products used.

	Mean	Standard deviation
Egg Weight [g]	68.1	2.37
Egg Height [mm]	58.3	1.03
Egg Diameter [mm]	45.6	0.72
Tomato Weight [g]	82.1	19.57
Tomato Height [mm]	47.8	4.05
Tomato Diameter [mm]	53.4	3.46

### 3.3 Tastemaps

**FIGURE 3:**
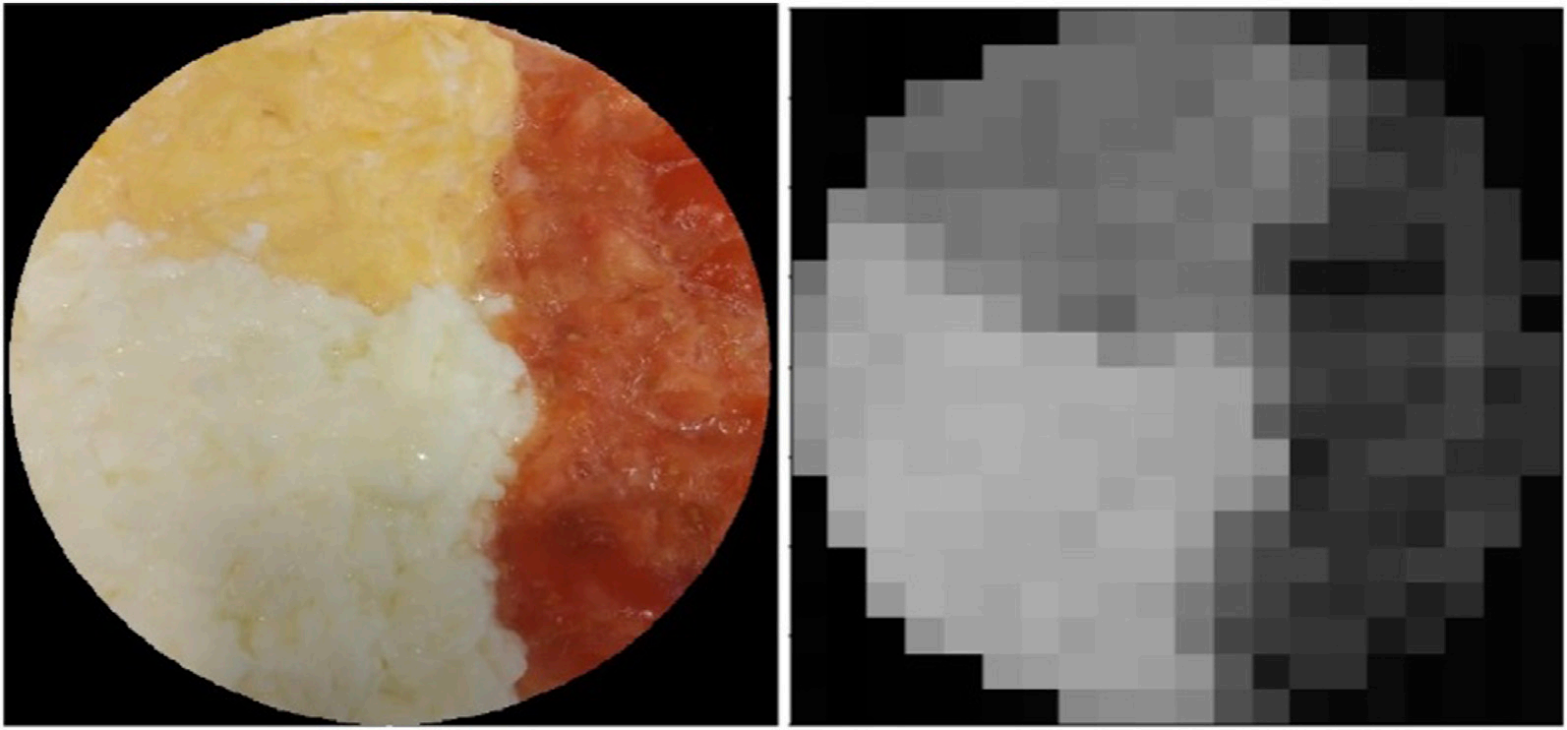
Trivial meal (left) and it is taste map (right) produced with a salinity sensor. The meal is made out of unsalted scrambled eggs, scrambled egg whites, and blended tomatoes, placed separately.

The taste information acquired during the experiments can be mapped and shown as an image. Using an image enables effortless understanding of the data for humans. Our setup produces taste maps based on two parameters–the number of test points and plate size. The test points are placed on a square grid, which is generated by constructing a square around the plate with a side length equal to the plate’s diameter. Then a required number of test points is distributed evenly inside the square. This number is limited to squares of integers to ensure that test points are placed on a square grid. In the next step, the check is done which of the points lay inside the plate, rather than in the part of the square outside the plate. Only points inside the plate are sampled, while the value of all other points is set to 0. An example of this method at work is shown in Figure 3, where we map a trivial meal composed of unsalted scrambled eggs, unsalted scrambled egg whites, and blended tomatoes using this method. These measurements are further used for taste metrics extraction.

### 3.4 Classification Task

**FIGURE 4:**
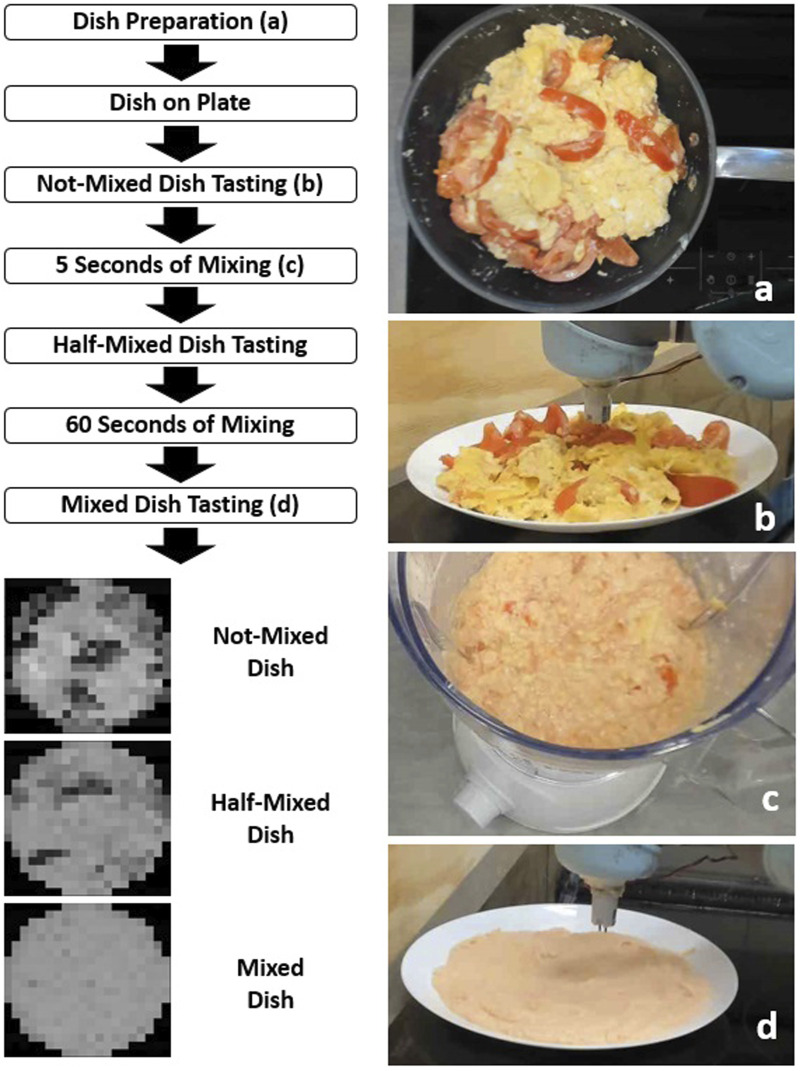
The experimental procedure used to taste a dish at various stages of chewing. **(a)** the dish is prepared according to a recipe. **(b)** robot sampling unmixed dish. **(c)** mastication is represented by mixing. **(d)** robot sampling mixed dish.

We set up a classification task to evaluate each of the tasting methods. We prepare nine variations of scrambled eggs with tomatoes for the experiment. These are made by adding three different amounts of tomatoes and three different amounts of salt to a fixed base of 6 large eggs. The tomato amount is set to 0, 3 or 6 tomatoes. The salt levels are 0, 1.2 and 2.4 g. Each combination of these levels of additives was used to cook a dish, resulting in 9 dishes, as detailed in Table 2. Each of the dishes is treated according to the experiment procedure shown in Figure 4. Cooking starts with placing 6 eggs in a pan, then salting them evenly with a prescribed amount of salt. After that, each tomato is cut into 8 sections and added to the dish. The dish is heated on the hob until the eggs scramble while mixing slowly, but constantly resulting in smooth eggs. These actions are done by a human, but constant mixing was done to reduce bias from human cooking and improve repeatability. Further, the dish is left to cool down to room temperature, to avoid the effect of temperature as an additional experimental condition. In real-world scenarios, the temperature effect on conductance can be compensated for, which is beyond of the scope of the current study. After cooling, the first tasting is done, with 400 samples spread across 16 × 16 cm square. This number is a result of a trade-off between time required for experimentation and producing a large enough dataset, with 400 samples being enough for training and collecting more is made difficult by the probe size (around 1 cm spacing between the electrodes). Out of these 400 samples, 324 lie inside the plate and become test points. Each dish is tasted three times, but only the first and the last tasting is used for classification to improve experiment repeatability. The first tasting is done on not mixed food, giving an experience right at the beginning of the chewing process. Then, the sample is mixed for a few seconds and is tasted again. This measurement is used for visualization only. After another 60 s of mixing on maximum RPM, the dish is tasted again. This amount of mixing is more than sufficient for the dish to become a homogeneous pulp well before the end of the process, hence making the procedure easy to replicate. This final tasting measures the flavour of the dish during the final stages of chewing.

**TABLE 2:** Table showing composition of 9 dishes used in classification experiment. The listed ingredients are combined with 6 large eggs to make a tomato scramble.

		Amount of Tomato		
		No tomato	Medium tomato	High tomato
		(0 tomatoes)	(3 tomatoes)	(6 tomatoes)
**Amount of salt**	**No salt (0g)**	Dish 1	Dish 2	Dish 3
		No Salt	No Salt	No Salt
		No Tomato	Medium Tomato	High Tomato
	**Medium salt (1.2g)**	Dish 4	Dish 5	Dish 6
		Medium Salt	Medium Salt	Medium Salt
		No Tomato	Medium Tomato	High Tomato
	**High salt (2.4g)**	Dish 7	Dish 8	Dish 9
		High Salt	High Salt	High Salt
		No Tomato	Medium Tomato	High Tomato

Furthermore, the data is processed to produce multiple instances for each of the classes. Each class corresponds to one of the dish types (Dish 1–9). The instances of the classes are subsets of samples collected tasting a dish of a specific class. This instance or subset represents a real-world tasting, that is done with a smaller amount of samples. This procedure enables leveraging data collected from a single dish for each of the classes. Therefore, 40 instances of each class were made, each containing randomly chosen 40% of the samples collected in the tasting. Each of the resulting instances contains 129 samples collected before chewing and the same number of samples collected after chewing. The resulting instances are then split into training and testing data sets at a 4:1 ratio. Next, each instance is reduced to a set of 4 numbers–mean and variance, both before and after chewing. These are the inputs to the SVM classifiers. SVM was chosen due to its good performance with limited data. This is crucial as acquiring data is extremely hard when working with food due to the cost and time involved in cooking. This fact makes approaches like neural networks or reinforcement learning impossible to apply.

**FIGURE 5:**
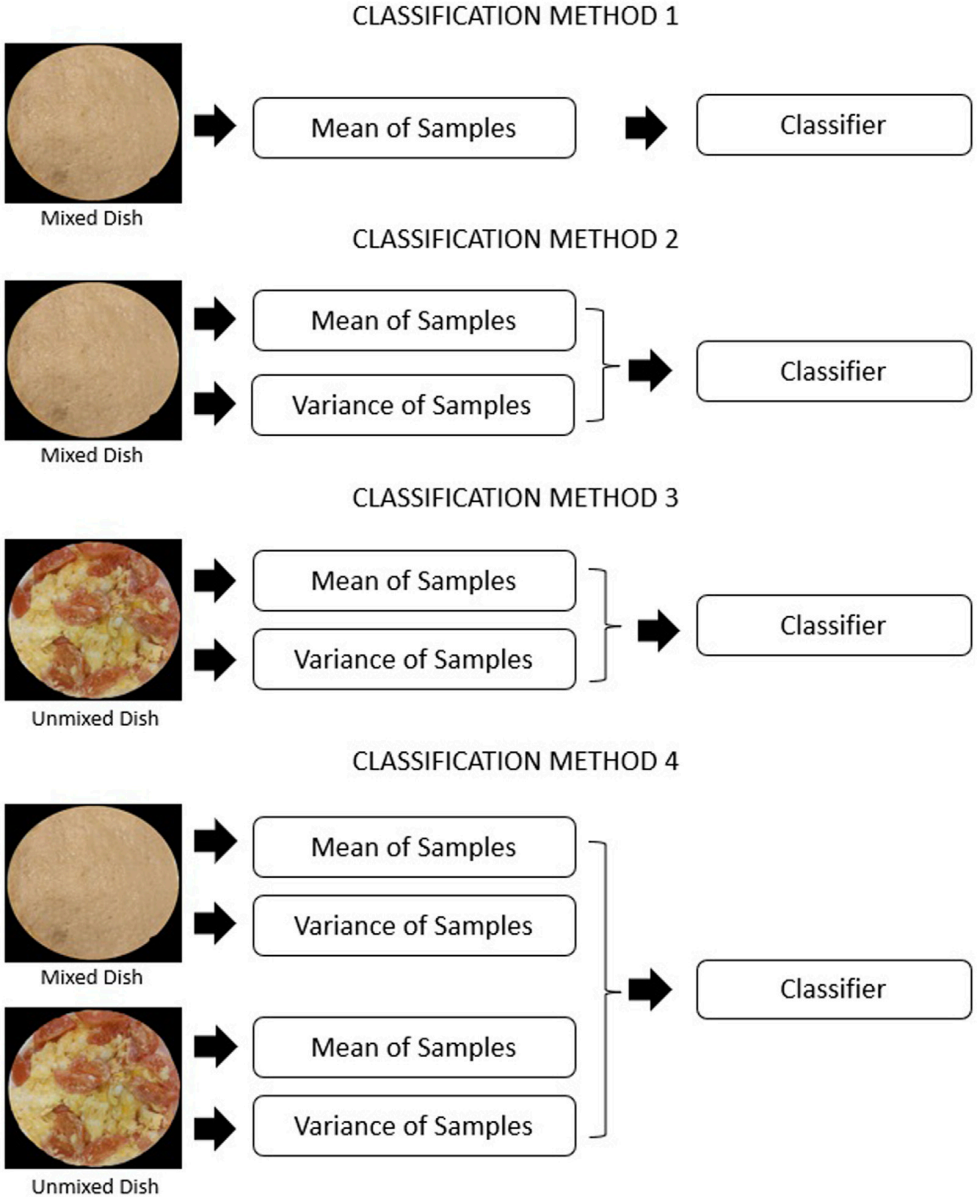
Schematics showing classification methods tested. Each dish was tasted twice–before and after chewing, with taste metrics extracted for at each stage.

We test a few methods of classification. All of them use an SVM classifier with the same settings, that performs one-vs-many classification for each of the classes. We did not see a noticeable difference in classifier performance varying penalty factor C in range 0.8–1.2. Therefore, default value of 1 was used for all the experiments. We use a polynomial kernel and balance classes weights to correct for an uneven number of samples, caused by the one-vs-many approach. The methods differ only by the type of data available to the classifier, but it introduces a profound change. From the point of view of the classifier, it brings additional data about every class, and this data add additional dimensions to the space where the SVM is finding a boundary between the classes. Each of the configurations represents a different method of tasting, even if all the data is gathered in a single experiment where the chewing is performed in the most general way. The configurations are shown in Figure 5.

The first configuration recreates the current state of the art method of tasting a homogeneous pulp. It is done by computing a mean of the readings from a mixed dish, which is an approximation of a perfectly homogenized sample. The second and third configuration takes both taste metrics–taking the advantage of the spatial distribution of taste–from either mixed or unmixed dish. It simulates the tasting only at one moment of the mastication process. The last configuration uses both metrics, calculated both before and after mixing to make a classification. It is a configuration that is closest to natural tasting and implements the tasting approach shown in Figure 1.

## 4 Results

### 4.1 Quantitative Representation of Taste

**FIGURE 6:**
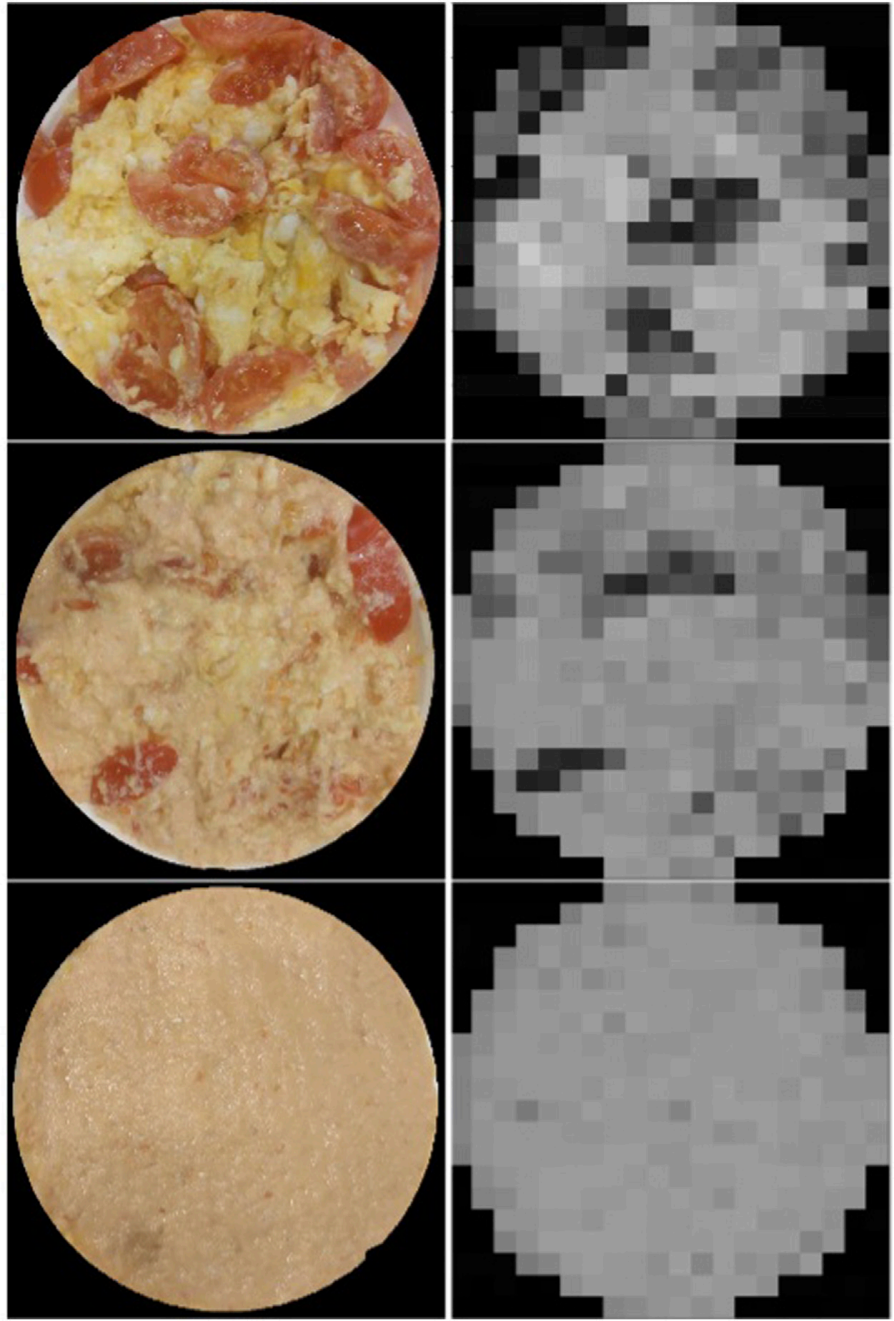
Figure showing the taste mapping of the same tomato scramble after mixing it to three different stages, with unmixed and “visually homogeneous” being the extreme cases.

In this section, we construct taste maps of one of the dishes at different stages of mastication. The resulting maps, together with a picture of the tasted dish are shown in Figure 6. The unmixed sample shows many very distinct areas of lowered conductivity in the meal, with very sharp borders visible between these and scrambled eggs. The next sample–half-mixed - shows less of these regions, and those still present are less sharply defined. Moreover, the scrambled eggs “area” itself became less conductive, possibly due to tomato juice being mixed in into this area. Finally, the last sample shows a rather homogeneous conductance distribution, with a conductance value in between the conductance of the tomato and the eggs. All spatial information is erased, producing pulp similar to those used for electronic tongues currently. Moreover, each of the stages of mastication results in a significantly different taste map, therefore providing additional information.

### 4.2 Effects of Mastication

**FIGURE 7:**
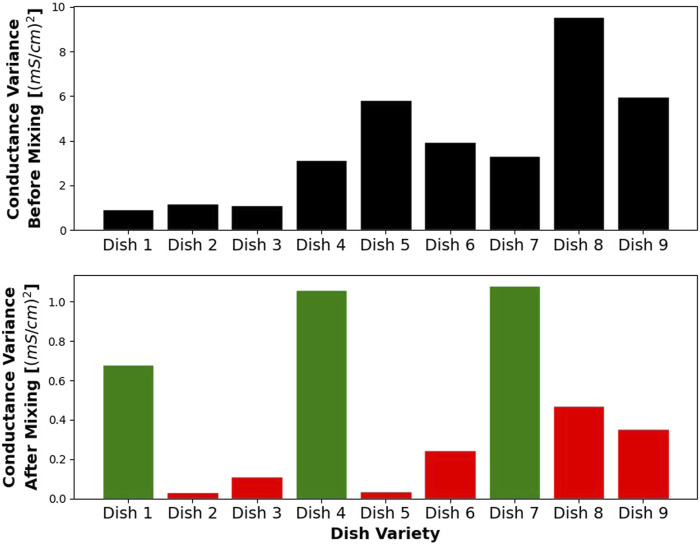
Figure showing the variance of salinity measurements, before and after mixing, for each dish. Dishes including tomato (red) tend to have much lower variance after mixed.

Variances of conductance measurements for each of the dishes are presented in Figure 7. Analyzing them showed that adding the tomato significantly decreases post-chewing variance. Running multivariate linear regressions shows that adding tomato (any non-zero amount) lowers the post-chewing variance by 0.73 mS/cm and is statistically significant with a *p*-value of 0.000019. The amount of salt, another variable in this model had a much smaller effect size of 0.1 mS/cm and a *p*-value of 0.013. This shows that the addition of the tomato is indeed the dominant factor reducing the variance of measurements.

**FIGURE 8:**
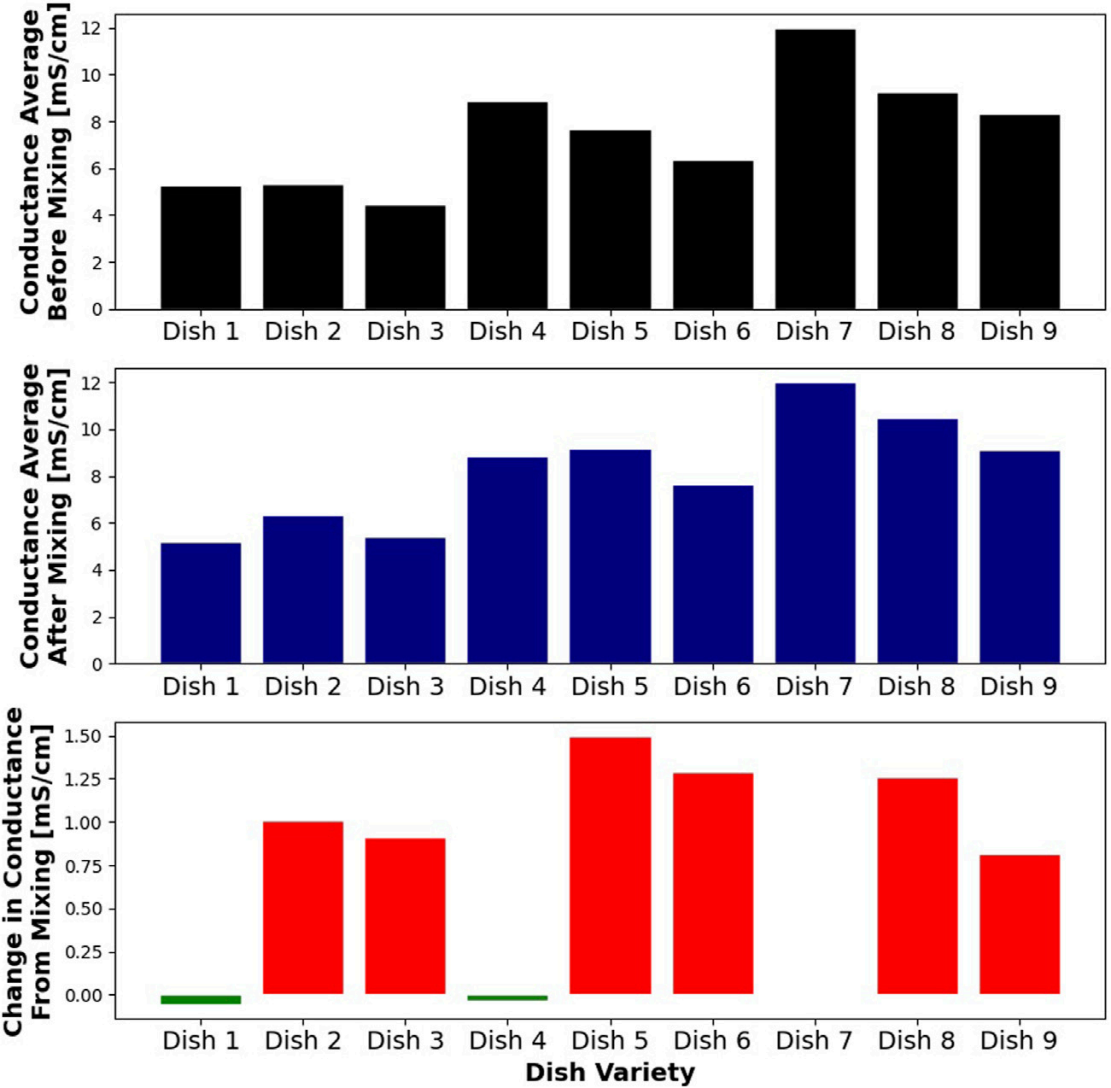
Figure showing the average salinity of gathered samples, before and after mixing, for each dish. Dishes including tomato (red) show an increase in the average salinity due to mechanical processing.

Another effect that we observed is the rise of conductance average during the chewing process, but again only in the cases when tomato was a part of the dish variance. The difference between post-chewing variance and pre-chewing variance for different dish varieties is shown in Figure 8. Matching the data to a multivariate linear model shows that adding tomatoes increases this value by 1.15 mS/cm, and it is statistically significant. Changing the amount of salt does not have a statistically significant effect.

**FIGURE 9:**
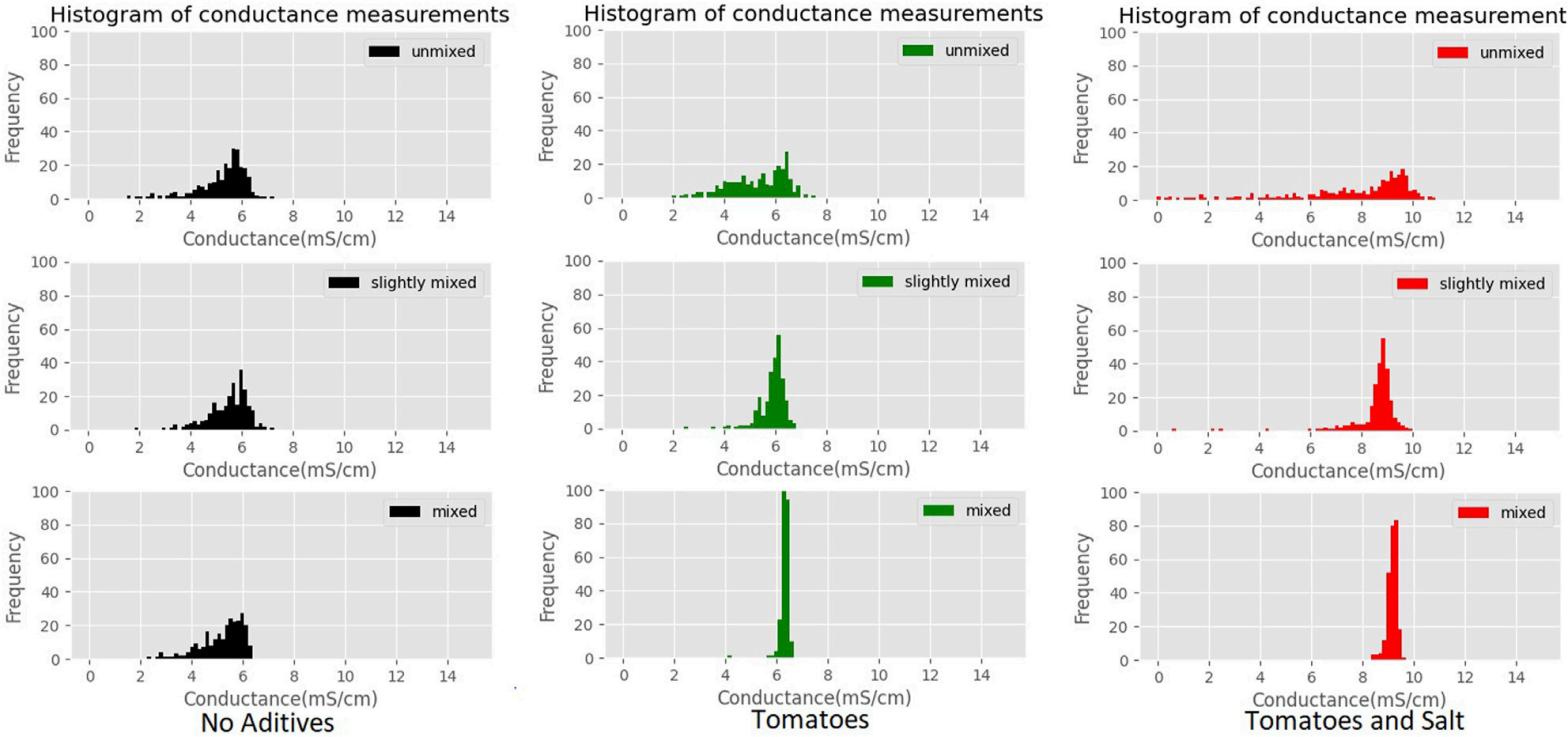
Histograms of conductance measurements at each of the mixing stages and with different mixtures of additives.

Histograms showing the effects of adding extra ingredients are shown in Figure 9. The dish without any additional ingredients does not change significantly when mixed. Mixing starts to have much more effects if a tomato is added, squeezing the histogram at later mixing stages. Finally, adding salt moves the histogram to the higher values, not changing its shape considerably.

### 4.3 Classification of the Dishes

**FIGURE 10:**
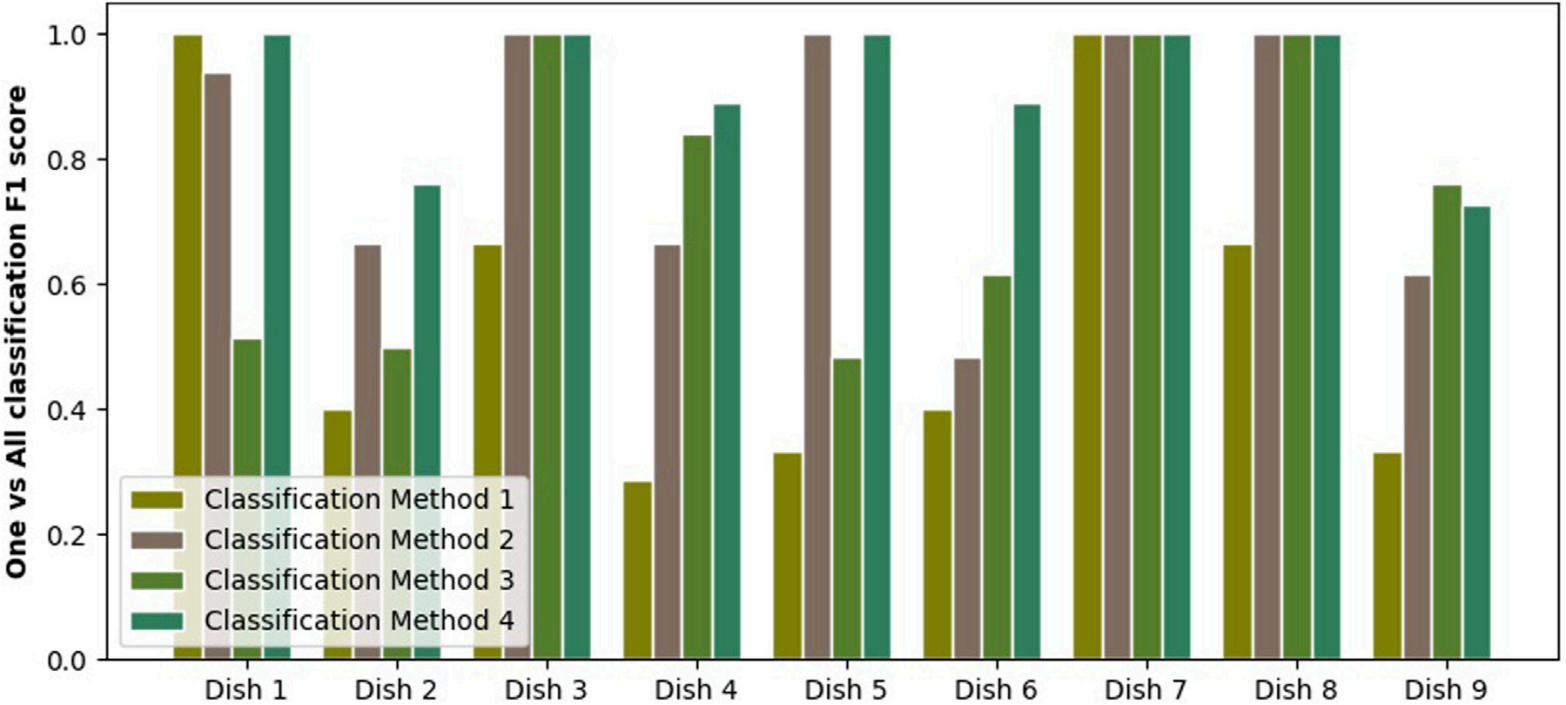
Figure showing a F1 score for one-vs-all classification done for each variation of tomato scramble. The classification was done based taste collected in four ways: homogenized sample, mixed sample, unmixed sample and unmixed and mixed sample together.

**FIGURE 11:**
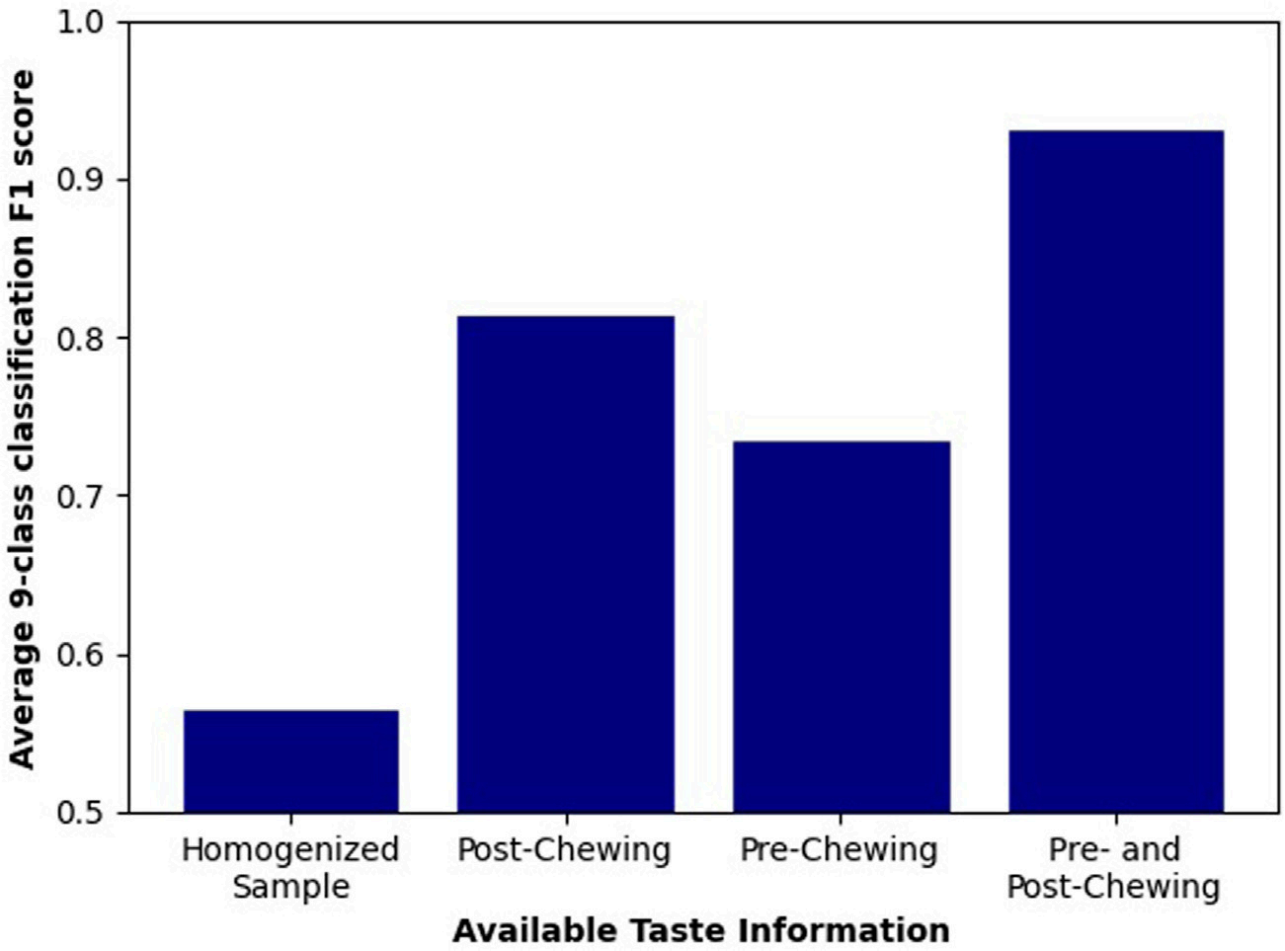
F1 score averaged from dish specific classifications. It shows a steady growth of F1 score while additional information is introduced.

**FIGURE 12:**
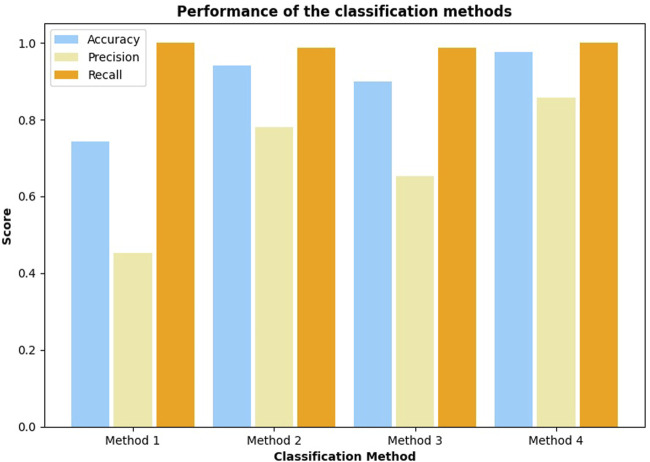
The figure shows accuracy, precision and recall, averaged across all dishes. Precision is the main parameter that improves with more information provided.

The quality of classification is measured using the F1 score, as it punishes both false negative and false positive errors when evaluating a classifier. We assume that both of these types of errors are equally undesirable, as we believe that both precision and recall are important in the future implementation of a robotic chef. Therefore, the F1 score is chosen as a single measure balancing the accuracy and recall in a single number. Results of a one-vs-all classification for each of scrambled eggs variations are shown in Figure 10. Each of the four configurations of tasting is shown as a separate bar. We can see that variations are easier to classify than others, but we also see that overall classification quality is rising with the introduction of extra information. Clearer trends are extracted by looking at the average F1 scores, which are shown in Figure 11. We clearly see the first method (homogenized sample tasting) comes with the lowest result while the last method (tasting both before and after chewing) scores the highest. Other methods come with medium scores. Moreover, we investigate the results further. Figure 12 shows the accuracy, precision and recall of each of discussed classification methods. Firstly, we see that accuracy varies between 75% and 95% and follows a trend similar to the previously shown F1 score. Recall, on the other hand, is always very high, showing that the classifier almost always recognizes the dish of the tested class. Furthermore, precision closely follows the same trend as the F1 score, and due to consistently high recall. Therefore, we can conclude that the performance improvements come from improving the precision and reducing the number of false-positive classifications. We speculate it is due to our tasting method effectively adding extra features and placing the dishes in a space of higher dimensionality, making it easier for the SVM to classify.

## 5 Discussion and Conclusion

### 5.1 Tasting Configurations’ Performance

While some of the results seem intuitive, like for example the homogenized sample performing the worst, there are lots of effects that were dish specific or pose an interesting question for future research. Starting from the homogenized sample–it performed the worst–probably because, in some cases, it faced an impossible task. It is because mixing different amounts of salt and tomatoes could bring the same average salinity. Therefore, due to specific sensor construction, this tasting mode results in the same readings for different ingredients mixtures. This applies to almost all of the current implementations of an electronic tongue, which are hopeless to distinguish between two dishes of the same chemical composition. This is a serious limitation especially if implemented tongue measures a limited number of substances.

**FIGURE 13:**
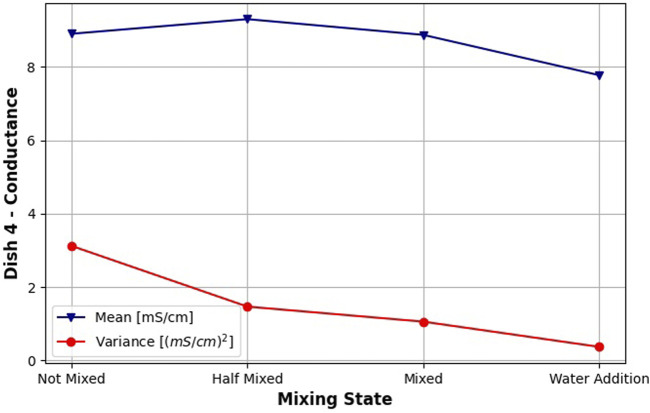
Effect of adding water to a dish variety with no tomato. Mean and variance of salinity measurements for three different stages of chewing and additional mixing after adding 50 ml of tap water.

Tasting a chewed dish performed surprisingly well given a very small difference in tasting procedure between it and the homogenized dish tasting. We believe it deserves some investigation. We notice that the dish variations without tomato have a variance of around 1 mS, while the dishes containing tomato tend to have their variance to fall to almost 0. We believe this is the effect that allowed differentiation between dishes of very similar average salinity. We attribute it to tomato juice released while chewing, moisturizing the dish during mixing and preventing the formation of air gaps in the dish. We confirm this theory by experimenting once again on the dish containing a middle salt level and no tomato. This time we taste it at 4 different points. Two of them are our standard before and after mixing tastings. We add tasting in the middle of mixing (same as for taste maps generation). Also at the end of the experiment, we add around 50 ml of tap water to the sample and mix it further. We use Dish 4 for this purpose, because it is one of three dishes for which this experiment is valid (dishes with no tomato), and it is the dish with a medium amount of salt. Therefore, we think it is the most representative dish for this kind of experiment. We plot the mean and variance of this experiment in Figure 13. The addition of the water enabled the fall of the variance to virtually zero, as in all dish variations that included tomato. It shows that the taste perceived by the robot can be affected by dry samples, as it doesn’t moisturize the sample as it is naturally done by saliva.

Tasting a not chewed sample seems to underperform in comparison with the previous sample, even if according to taste maps it seems to contain more information. We believe it is due to the very simple algorithm we use for classification. All spatial information is compressed to a single number by computing variance. Perhaps, taste maps could be processed in the future similarly to pictures–using computer vision. Convolutional neural networks may also find use in the future, but only if making large data sets becomes feasible.

Tasting at both stages of mastication performed the best, which is not surprising. This is because tasting at different stages of mastication provides different information, and this configuration has access to all information available to all other configurations. Therefore, it can increase the classification quality by spreading the data into more dimensions, making it easier for SVM to find a boundary between them.

### 5.2 Limitations

While a human is continuously tasting while chewing, acquiring the information at a large number of chewing stages is hard to recreate with the proposed setup. This is due to the lack of controllability of the mixing process that makes it impossible to apply exactly the same mixing to each of the tasted dishes. We work around this limitation by limiting the number of tasting to 2. The sensing is therefore done on not chewed dishes and then on a dish fully chewed. Fully chewed dishes are processed significantly longer after the dish looks homogeneous. Therefore, we apply the maximum possible mixing, that is easy to replicate. Future work should explore the possibility of sampling at more stages. Moreover, the strong temperature dependence requires cooling down the dish to room temperature before sensing. Even though it effectively keeps the temperature stable, it stops the robot from tasting during cooking. Implementation of feedback during cooking would be a huge step towards matching human cooking skills with a robot.

It is reasonable to expect that the proposed approach with mastication works best for solid-state non-homogeneous dishes, especially if they contain a significant amount of water. This group contains all stews, soups, scrambled eggs with additives, salads or baked beans. Moreover, it is the only known approach that may taste a full course, made of main and sides, where the spatial distribution of the ingredients matter. On the other hand, some dishes like liquids and yoghurts may benefit from the proposed approach less, due to little effect of chewing on these foods.

### 5.3 Conclusion and Future Work

In the paper, we simulated chewing with a robotic setup and used it to extract additional information by taking spatially and temporally separated samples. We introduced taste maps as a visualization tool, that proved that additional, non-trivial information is present at each stage of chewing. We show that imitation of natural mastication results in higher classification performance than tasting homogenized samples. We also investigate some phenomena contributing to these changes like the role of moisturizing the sample.

Future work should include an investigation of saliva as part of the robotic tasting, perhaps including chemical reagents to recreate lipase and amylase present in human saliva. Moreover, we will investigate the usefulness of the proposed approach for other dishes in the future. Finally, we want to investigate the fundamental question of how we approach recreating the taste, how we process it and what form the taste output fundamentally is. We should also consider if classification is a good approach to tasting or should we long for measuring exact chemical composition as an analogue value. Considering the lowest level–a single taste receptor on the tongue–it is recording an analogue signal, but the signal finds its way to our consciousness in a completely different form. Therefore, on the higher abstraction level–the psychophysical approach–classification tasks are frequently used working with human participants (e.g., asking which of the two samples is saltier). Furthermore, looking at the basic evolutional role of taste–bringing information to enable a decision if to eat something–the taste is used for a classification task. Finally, if we think about the enjoyment we gain from eating we see it as an analogue signal, yet again it is not a solid number like a chemical composition analysis. Therefore, currently, we are using classification as a convenient benchmark and as a benchmark that can be used in the future to compare robotic taste to human taste psychophysical studies, while we believe this concept should be extended in the future.

## Data Availability

The datasets presented in this study can be found in online repositories. The names of the repository/repositories and accession number(s) can be found below: https://github.com/Grzegorr/Paper-Taste-Mastication/tree/main/PythonCode/Data.
